# Amisulpride withdrawal akathisia responding to aripiprazole with propranolol in first-onset psychosis: a case report

**DOI:** 10.1186/s12888-022-03721-9

**Published:** 2022-01-29

**Authors:** Hae-Jung Yang, Seung-Gon Kim, Eun Hyun Seo, Hyung-Jun Yoon

**Affiliations:** 1 Department of Psychiatry, Chosun University Hospital, Gwangju, Republic of Korea; 2 Department of Psychiatry, College of Medicine, Chosun University, 309 Pilmun-daero, Dong-gu, Gwangju, 61452 Republic of Korea; 3 Premedical Science, College of Medicine, Chosun University, Gwangju, Republic of Korea

**Keywords:** Amisulpride, Aripiprazole, Withdrawal, Akathisia, Hyperprolactinemia

## Abstract

**Background:**

Akathisia tends to develop as an early complication of antipsychotic treatment in a dose-dependent manner. Although withdrawal akathisia has been reported after the discontinuation or dose reduction of typical antipsychotic drugs, akathisia following atypical antipsychotic drug withdrawal remains a rare phenomenon.

**Case presentation:**

A 24-year-old woman with an acute psychotic episode was admitted and initially treated with aripiprazole. The aripiprazole dose was titrated up to 30 mg/day over 9 days and maintained for the next 3 days; however, her psychotic symptoms persisted without change. She was switched to amisulpride, with the dose increased over 2 weeks to 1000 mg/day. Subsequently, although the patient’s psychotic episode subsided, her serum prolactin levels increased markedly. After discharge, the amisulpride dose was increased to 1200 mg/day owing to auditory hallucinations and was maintained with quetiapine (100–200 mg/day) and benztropine (1 mg/day) for 13 weeks. Given the potential for hyperprolactinemia as a side effect, the amisulpride dose was reduced to 800 mg/day concurrently with the discontinuation of benztropine; however, these changes resulted in severe restlessness without other extrapyramidal symptoms. The withdrawal akathisia disappeared over 2 weeks after switching to aripiprazole (10 mg/day) with propranolol (40 mg/day) and the patient’s prolactin levels had normalized after 6 months of aripiprazole monotherapy.

**Conclusions:**

The present case highlights the potential for the development of withdrawal akathisia when the dose of amisulpride is tapered abruptly. Thus, a slow tapering and careful monitoring are recommended when switching from amisulpride to other antipsychotic drugs. Furthermore, this case suggests that changing the regimen to aripiprazole with propranolol may be a potential option for amisulpride withdrawal akathisia superimposed on pre-existing hyperprolactinemia.

## Background

Akathisia is a movement disorder characterized by a subjective sense of inner restlessness and objective restless movements, and is commonly associated with the use of typical antipsychotic drugs [1]. Early detection and treatment of akathisia are important as the condition has been associated with nonadherence, treatment-emergent suicidality, and violent behaviors in patients with schizophrenia [2, 3]. Generally, akathisia tends to occur in a dose-dependent manner as an early complication of antipsychotic drug treatment. It has also been reported after the discontinuation or tapering of typical antipsychotic drug dose [4, 5]. However, atypical antipsychotic withdrawal akathisia remains a rare phenomenon. Here, we report the first case of amisulpride withdrawal akathisia, which was controlled by aripiprazole with propranolol.

## Case presentation

A 24-year-old unmarried woman was admitted to the inpatient psychiatric ward at a teaching hospital because of an insidious onset of auditory hallucinations, blunted affect, alogia, abulia, and negativism that had begun approximately 1 month previously. She had no personal or family history of psychiatric illness, and this was her first psychotic deterioration. The diagnosis of schizophreniform disorder was established according to the fifth edition of the Diagnostic and Statistical Manual of Mental Disorders, supported by normal laboratory test results, including electroencephalography and brain magnetic resonance imaging. On the first day after admission, pharmacotherapy with aripiprazole was initiated at a dose of 10 mg/day, which was increased to 30 mg/day over 9 days. Quetiapine was administered at a dose of 100–200 mg/day at the same time for alleviation of insomnia. This treatment was well tolerated and maintained for the next 3 days; however, the patient’s psychotic symptoms persisted without any changes. Based on clinical judgment, aripiprazole was discontinued over 2 days. Simultaneously, amisulpride was started at a dose of 100 mg/day and increased to 1000 mg/day along with quetiapine (200 mg/day) for 10 days. During the titration of amisulpride, benztropine (1 mg/day) was added because the patient complained of subjective discomfort in her right lower extremities. From the 22nd day of admission, her psychotic symptoms improved, as verified by a significant reduction in the scores of objective symptom rating scales (Table 1). However, her serum prolactin level had markedly increased to above 200 ng/mL on the 22nd day, compared to 5.66 ng/mL on the 9th day of admission, before she had been administered amisulpride, indicating the development of amisulpride-induced hyperprolactinemia. On the 25th day of admission, the patient was discharged.

**Table 1:** Change in the scores for psychiatric symptom rating scales during admission

	3th day	22th day
BPRS	41	28
PANSS		
Total	92	49
Positive subscale	21	13
Negative subscale	24	12
General psychopathology subscale	47	24

*BPRS* Brief Psychiatric Rating Scale, *PANSS* Positive and Negative Syndrome Scale

Eight days after discharge, the patient visited an outpatient clinic and reported that she was fine except that her auditory hallucinations sometimes worsened. Thus, amisulpride was increased to 1200 mg/day, which was maintained with quetiapine (100–200 mg/day) and benztropine (1 mg/day) for 13 weeks. This treatment was effective and safe and her auditory hallucinations nearly disappeared. Although the patient did not experience any hyperprolactinemia-related symptoms, such as amenorrhea, the hyperprolactinemia was highly likely to persist owing to the higher dose of amisulpride than that used during admission. Given the potential complications with long-term amisulpride use, including osteoporosis and cardiovascular diseases, the dose was reduced to 800 mg/day directly. Concurrently, benztropine was discontinued, and quetiapine (100 mg/day) was maintained. After 4 weeks, she reported the development of severe restlessness over the previous 3 weeks, which made her want to walk around her room. In the outpatient clinic, she complained of a severe compulsion to move and an inability to sit and continued to walk from side to side during the interview. Her Barnes Akathisia Rating Scale score was 6. The prior regimen was changed to aripiprazole (10 mg/day) with propranolol (40 mg/day) without a period of overlap. Since then, the patient’s restlessness greatly improved over 2 weeks. After withdrawal, the akathisia disappeared, propranolol was discontinued, and aripiprazole monotherapy (10 mg/day) was maintained. The patient has remained stable without any signs of extrapyramidal symptoms (EPS) or psychotic episodes for 6 months and her prolactin level has normalized (4.55 ng/mL).

## Discussion and conclusions

Although atypical antipsychotic drugs are known to have lower risks of EPS than typical antipsychotic drugs, recent reviews have shown that atypical antipsychotic drugs are not free from causing akathisia, with composite rates ranging from 2.9 to 13.0% [6]. The prevalence of akathisia varies across atypical antipsychotic medications, with risperidone and aripiprazole showing the highest and quetiapine and iloperidone the lowest prevalence [7]. Meanwhile, studies on amisulpride-induced EPS or akathisia are lacking. Few cases of amisulpride-induced tardive dyskinesia or dystonia have been reported [8, 9]. Lo and Peng [10] described a case of severe withdrawal dyskinesia, along with dystonia and akathisia, after the dose of amisulpride was reduced from 200 to 50 mg/day. The abnormal involuntary movements improved 2 weeks after the dosage was increased to 100 mg/day. However, amisulpride withdrawal akathisia without any other signs of EPS has not yet been described. The present case suggested that severe withdrawal akathisia could occur when amisulpride is tapered abruptly. Thus, a slow tapering and careful monitoring for potential akathisia are needed when switching from amisulpride to other antipsychotic drugs.

Amisulpride has a selective affinity for dopamine D_2_/D_3_ receptors in the limbic regions compared to the striatal regions. A high dose of amisulpride preferentially blocks postsynaptic D_2_/D_3_ receptors, whereas low doses block presynaptic D_2_/D_3_ receptors, resulting in enhanced dopamine transmission [11]. Despite the lack of 5-HT_2A_ antagonism, amisulpride is classified as an atypical antipsychotic, which could be attributed to its preferential action on limbic D_2_/D_3_ receptors. Although the pathophysiology of akathisia is not yet fully understood, Ferré et al. [12] recently proposed that the common mechanism of akathisia involves increased presynaptic dopaminergic transmission in the ventral striatum and a concomitant activation of the D_1_ receptor localized in the ventral GABAergic striatonigral neurons, which form heteromers with D_3_ and adenosine A_1_ receptors. In the present case, an abrupt amisulpride tapering may have led to changes in striatal D_2_/D_3_ receptor occupancy, thereby inducing a relatively hyperdopaminergic state. The akathisia improved after switching to aripiprazole with propranolol. In addition to ventral striatal dopamine release, hyperserotonergic neurotransmission and increased central norepinephrine activity have also been implicated in akathisia [13, 14]. Aripiprazole acts as a partial agonist of D_2_/5-HT_1A_ receptors and an antagonist of 5-HT_2A_ receptors [15]. The dopamine and serotonin stabilizer function of aripiprazole combined with the additional reduction of noradrenergic neurotransmission by propranolol may explain the anti-akathisia effects observed in our case.

Antipsychotic-induced hyperprolactinemia can cause menstrual irregularities, amenorrhea, galactorrhea, and sexual dysfunction in the short term, negatively impacting drug compliance as well as the risk of tumorigenesis and osteoporosis in the long term [16]. Thus, early detection and appropriate management of antipsychotic-induced hyperprolactinemia are important for the treatment of schizophrenia spectrum disorders. As in our case, amisulpride can induce a marked elevation of prolactin level, similar to that of typical antipsychotics, which is often clinically relevant [17]. Contrary to other antipsychotic drugs that block the D_2_ receptor on lactotroph cells, aripiprazole showed a mixed agonist/antagonist profile, depending on the preexisting dopaminergic tone [18]. Chen et al. [19] reported that adjunctive treatment with aripiprazole effectively reversed hyperprolactinemia induced by risperidone, but not by benzamide antipsychotic drugs, such as amisulpride. Amisulpride reaches higher concentrations in the pituitary gland because of its poor blood-brain barrier penetration [20], which explains why aripiprazole add-on cannot reverse amisulpride-induced hyperprolactinemia. Hence, we switched the patient to aripiprazole from amisulpride, which resulted in the normalization of her serum prolactin level.

This report has several limitations. First, it is difficult to completely rule out the effect associated with the discontinuation of benztropine, such as cholinergic rebound. Second, amisulpride-induced delayed onset akathisia is also a possibility owing to its delayed penetration into the brain [21]. Third, we did not attempt to increase the dose of amisulpride. Alleviation of the patient’s restlessness following the dose increase would have helped to further confirm the withdrawal akathisia. Fourth, given the prior poor response and relatively higher incidence of akathisia, other atypical antipsychotics such as quetiapine, rather than aripiprazole, may have been a better choice to manage akathisia as well as the symptoms of schizophreniform disorder. Aripiprazole may cause akathisia or an akathisia-like syndrome but is usually associated with a lesser weight gain than other atypical antipsychotics. The patient was a young woman and expressed concern regarding weight gain as a side effect of medication. Further, aripiprazole monotherapy has little effect on and may actually lower prolactin levels in individuals with prior antipsychotic exposure [22]. For these reasons, aripiprazole was restarted in our case. Finally, we did not perform dopamine imaging, which would have provided objective evidence for the underlying cause of withdrawal akathisia.

In summary, we have described the first case of severe akathisia following the tapering of amisulpride dose, which was successfully managed with aripiprazole combined with propranolol. The present case emphasized the potential for the development of withdrawal akathisia after an abrupt reduction of amisulpride dose. Hence, a gradual tapering and careful monitoring for akathisia and EPS are recommended when changing from amisulpride to other antipsychotic drugs. Furthermore, this case suggested that switching the regimen to aripiprazole with propranolol may be considered a potential option for amisulpride withdrawal akathisia superimposed on pre-existing hyperprolactinemia.

## Data Availability

Not applicable.

## Abbreviations

5-HT: 5-Hydroxytryptamine
EPS: Extrapyramidal symptoms
GABA: Gamma-aminobutyric acid
